# Next-Generation Sequencing of HIV-1 RNA Genomes: Determination of Error Rates and Minimizing Artificial Recombination

**DOI:** 10.1371/journal.pone.0074249

**Published:** 2013-09-18

**Authors:** Francesca Di Giallonardo, Osvaldo Zagordi, Yannick Duport, Christine Leemann, Beda Joos, Marzanna Künzli-Gontarczyk, Rémy Bruggmann, Niko Beerenwinkel, Huldrych F. Günthard, Karin J. Metzner

**Affiliations:** 1 Division of Infectious Diseases and Hospital Epidemiology, University Hospital Zurich, University of Zurich, Zurich, Switzerland; 2 Department of Biosystems Sciences and Engineering, ETH Zurich, Basel, Switzerland; 3 Institute of Medical Virology, University of Zurich, Zurich, Switzerland; 4 Functional Genomics Center Zurich (FGCZ), University of Zurich, ETH Zurich, Zurich, Switzerland; 5 SIB Swiss Institute of Bioinformatics, Basel, Switzerland; McGill University AIDS Centre, Canada

## Abstract

Next-generation sequencing (NGS) is a valuable tool for the detection and quantification of HIV-1 variants *in vivo*. However, these technologies require detailed characterization and control of artificially induced errors to be applicable for accurate haplotype reconstruction. To investigate the occurrence of substitutions, insertions, and deletions at the individual steps of RT-PCR and NGS, 454 pyrosequencing was performed on amplified and non-amplified HIV-1 genomes. Artificial recombination was explored by mixing five different HIV-1 clonal strains (5-virus-mix) and applying different RT-PCR conditions followed by 454 pyrosequencing. Error rates ranged from 0.04–0.66% and were similar in amplified and non-amplified samples. Discrepancies were observed between forward and reverse reads, indicating that most errors were introduced during the pyrosequencing step. Using the 5-virus-mix, non-optimized, standard RT-PCR conditions introduced artificial recombinants in a fraction of at least 30% of the reads that subsequently led to an underestimation of true haplotype frequencies. We minimized the fraction of recombinants down to 0.9–2.6% by optimized, artifact-reducing RT-PCR conditions. This approach enabled correct haplotype reconstruction and frequency estimations consistent with reference data obtained by single genome amplification. RT-PCR conditions are crucial for correct frequency estimation and analysis of haplotypes in heterogeneous virus populations. We developed an RT-PCR procedure to generate NGS data useful for reliable haplotype reconstruction and quantification.

## Introduction

Human immunodeficiency virus type 1 (HIV-1) is a highly diverse virus, not only on a global scale, but also within individual HIV-1 infected subjects [1]. The genetic variants constituting the viral population are called haplotypes, and these haplotypes form a viral quasispecies [2]. It has been shown that low-abundant haplotypes are already present in patients shortly after infection [3]–[6]. Numerous studies have shown that minority drug-resistant HIV-1 variants can be clinically relevant and lead to therapy failure, especially in the context of pre-existing minority variants harbouring resistance mutations to non-nucleoside reverse transcriptase inhibitors (NNRTI) [7]–[10].

Viral diversity has major implications on pathogenesis, drug resistance, and vaccine development. Since next-generation sequencing (NGS) platforms are widely available, virus populations can be studied much faster compared to the classical methodology of single genome sequencing. However, these technologies require rigorous estimation of error rates and identification of error sources, especially when viral haplotypes are quantified (reviewed in [11]). For instance, several studies have investigated the accuracy of the pyrosequencing technology, and it is well known that homopolymeric regions are the main source of insertion-deletion (indel) errors [12], [13]. Moreover, the PCR polymerase can also contribute to this effect [14]. PCR artifacts are well known and addressed by optimizing PCR conditions and using high fidelity DNA polymerases [15]. Recently, primer identifiers have been described to circumvent some of the remaining PCR artifacts [16].

So far, not much attention has been drawn to the cDNA synthesis that is required as first step when RNA, rather than DNA, is the source for genetic analyses. RTs are error-prone enzymes [17], and misincorporations during cDNA synthesis are difficult to avoid and almost impossible to distinguish from real variations, especially in heterogeneous viruses such as HIV-1.

*In vitro* recombination has almost exclusively been studied on DNA templates and numerous improved PCR conditions have been described [18]–[28]. Amplifying a heterogeneous DNA sample can lead to artificial chimeras and therefore to an overestimation of genetic variation [18], [24], [25]. PCR-mediated chimeras are mainly created by prematurely terminated template extensions during PCR and subsequent false priming of these short sequences to a non-homologous sequence in the following cycles [21], [23]. A previous study has shown that PCR-induced recombinants can account for up to 30% of the final PCR product [19]. Several factors can influence PCR-induced *in vitro* recombination, including template amount and polymerase processivity [20]–[22], but *in vitro* recombination induced by reverse transcription is poorly studied. So far, only Fang and co-workers studied HIV-1 cDNA synthesis-induced *in vitro* recombination and showed that a 2.5-fold higher *in vitro* recombination rate can be observed in RT-PCR compared to DNA PCR when a long 4.5 kb fragment is amplified, probably due to prematurely terminated cDNA synthesis or RNA molecules degraded prior to the RT reaction [29].

Minimizing *in vitro* recombinants is particularly important when studying the intra-patient diversity of viruses like HIV-1. Besides a high mutation rate, this virus has the natural ability to recombine, which is one of several options of HIV-1 to circumvent selection pressures and to adapt to a new host [30], [31].

Here, we estimated the error rates and characterized possible error sources for the 454 pyrosequencing technology at all stages of the procedure. We established an optimized, artifact-reducing RT-PCR protocol to reverse transcribe, amplify, and pyrosequence HIV-1 RNA genomes enabling accurate haplotype analysis based on entire sequence reads.

## Results

### Substitution and Insertion/Deletion Rates and their Sources

**Figure 1:**
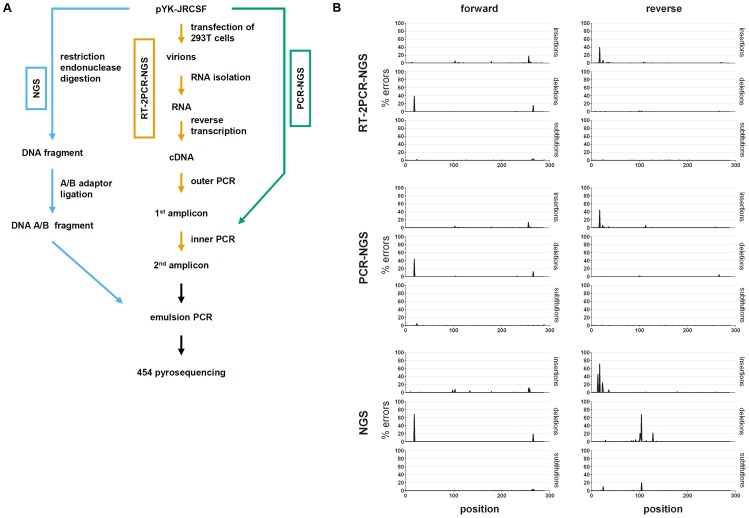
Substitution and insertion/deletion rates and their sources using 454 pyrosequencing. A) The molecular full-length HIV-1 clone pYK-JRCSF was used to generate three different samples for the determination of error rates and error sources during the different steps of sample preparation. The blue (left) pathway indicates the procedure NGS, i.e., no amplification step was performed before emulsion PCR and pyrosequencing. The green (right) pathway shows the procedure PCR-NGS, i.e., the target was amplified once prior to 454 emulsion PCR/pyrosequencing. The orange (middle) pathway depicts the commonly used procedure RT-2PCR-NGS to reverse transcribe, amplify and sequence HIV-1 RNA genomes reflecting the errors that will occur using patients’ plasma samples to analyze HIV-1 haplotypes. Detailed description of each step is given in the materials and methods section. B) Error rates per positions are shown for forward reads (left) and reverse reads (right). For each duplicate, one example is shown (always sample a as presented table 1).

To estimate the error rates of the different steps in the procedure of 454 pyrosequencing, the protease gene of the virus strain HIV-1_JR-CSF_ was amplified and 454 pyrosequenced following three different experimental procedures. In the first procedure, the plasmid pYK-JRCSF, containing the full-length sequence of HIV-1_JR-CSF_, was digested using restriction enzymes flanking the protease gene. Adaptors were ligated to the protease gene to obtain a fragment for direct 454 pyrosequencing. We refer to this sample as “NGS” (figure 1A). It is used to evaluate the substitution and indel (insertions and deletions) rates of the emulsion PCR and the pyrosequencing procedure. In the second set-up, the exact same plasmid preparation was used to amplify the protease gene using fusion primers that consist of a HIV-1 specific region, a multiplex identifier and either the A or B sequence required for 454 pyrosequencing. This sample is named “PCR-NGS”, as only one, the inner, PCR was done to obtain the amplicon (figure 1A). This experiment was performed to estimate the substitution and indel rates of PCR, emulsion PCR, and pyrosequencing. In the third set-up, again the same plasmid preparation was used to produce the virus stock HIV-1_JR-CSF_ from which viral RNA was isolated and reverse transcribed followed by outer and inner PCRs. This sample is named “RT-2PCR-NGS” (figure 1A). This set-up was used to estimate the substitution and indel rates of the complete procedure that is commonly applied to pyrosequence HIV-1 from patients’ plasma samples (RT, outer PCR, inner PCR, emulsion PCR, and pyrosequencing).

All three experimental procedures were set up in duplicates and pooled before pyrosequencing. Reads were aligned to the HIV-1_JR-CSF_ reference sequence, forward and reverse reads were analyzed separately (see Materials and Methods). Every difference between a read and the reference was counted as an error. Table 1 depicts the average substitution and indel rates per nucleotide for each sample. The substitution rates per nucleotide varied between 0.08–0.16%, not showing clear patterns in regard to either the different experimental procedures nor to forward and reverse reads. In contrast, indel rates varied considerably. In comparison, deletion rates were 2.7–5.5 -fold lower in reverse reads than in forward reads obtained from PCR-NGS and RT-2PCR-NGS samples and approximately twofold higher in reverse reads of NGS samples (table 1). Insertion rates varied less in forward and reverse reads of PCR-NGS and RT-2PCR-NGS samples, but they were >3-fold higher in reverse reads than in forward reads of NGS samples. The analysis of substitution and indel rates per position in forward and reverse reads revealed that these errors occurred mainly in the context of homopolymers (figure 1B). The longest homopolymer (six guanines) is located at position 18–23 (figure 1B). It mainly caused artificial deletions in forward reads and insertions in reverse reads, explaining the differences in average error rates (table 1).

**Table 1:** Substitution and insertion/deletion rates per base of different procedures for amplicon generation.

sample*	procedure	analysis strategy	totalreads	total readsanalyzed	total basesanalyzed	insertionrate [%]	deletionrate [%]	substitutionrate [%]
a	NGS	forward reads	3,898	1,463	421,588	0.19	0.31	0.06
b	NGS	forward reads	1,649	685	197,238	0.17	0.19	0.08
a	PCR-NGS	forward reads	50,173	21,214	6,106,238	0.16	0.22	0.14
b	PCR-NGS	forward reads	100,263	39,758	11,358,035	0.21	0.23	0.16
a	RT-2PCR-NGS	forward reads	37,724	16,798	4,842,383	0.19	0.20	0.11
b	RT-2PCR-NGS	forward reads	55,337	28,511	8,217,013	0.17	0.19	0.12
a	NGS	reverse reads	3,898	2,434	705,284	0.63	0.56	0.14
b	NGS	reverse reads	1,649	963	279,122	0.66	0.43	0.15
a	PCR-NGS	reverse reads	50,173	28,697	8,276,817	0.28	0.04	0.11
b	PCR-NGS	reverse reads	100,263	53,266	15,260,156	0.29	0.05	0.14
a	RT-2PCR-NGS	reverse reads	37,724	20,915	6,034,401	0.25	0.05	0.11
b	RT-2PCR-NGS	reverse reads	55,337	26,804	7,744,407	0.40	0.07	0.08

* each sample was done in duplicate, the sample name a and b helps to distinguish the duplicates from each other.

NGS, next-generation sequencing; RT, reverse transcription.

### Characterization of Molecular HIV-1 Clones

**Figure 2:**
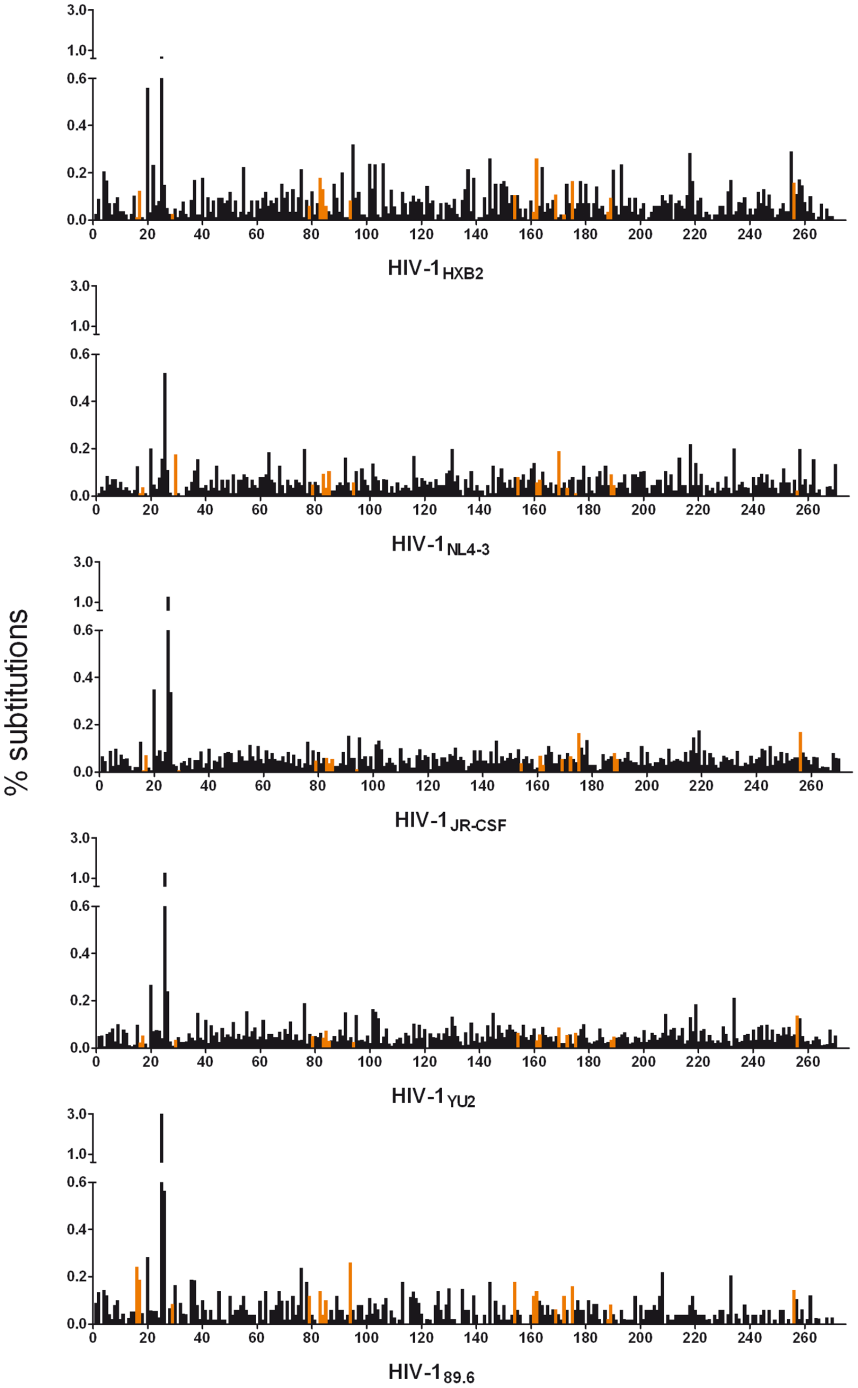
Substitution rates of each virus strain used to generate the 5-virus-mix. Each of the HIV-1 stocks was pyrosequenced separately to control for the purity of each virus strain. The y-axis shows the substitution rate per base according to the reference within the analyzed 271 bp long fragment (amino acids 10–99 of the HIV-1 protease, nt 2279–2549 based on HIV-1_HXB2_). The x-axis shows the positions on the sequence. The orange bars indicate differences in the nucleotide sequences of the five virus strains.

293T cells were transfected separately with five different HIV-1 full-length plasmids to obtain molecular HIV-1 clones. As a control, each of them was 454 pyrosequenced separately to estimate the substitution and indel rates and to exclude the presence of any recombinants in these virus stocks. Between approximately 5,000 and 34,000 reads were analyzed per sample. Within the 271 base pairs (bp) long analyzed region of the viral protease gene, the mean substitution rates were on average 0.09% per nucleotide and evenly distributed among the amplicons, except the high substitution rates within the six-G homopolymer region of the amplicons (figure 2). The insertion and deletion rates were on average 0.1% and 0.2% per nucleotide, respectively. Recombinants were not observed in any of these molecular clones.

Sequences were similar to the published sequences of each molecular HIV-1 clone except for HIV-1_89.6_. Here, our HIV-1_89.6_ strain consists of GAG instead of AGT at positions 2360-2 and of G instead of A at position 2371 (based on HIV-1_89.6,_ GenBank accession number U39362). The largest genetic distance was between HIV-1_NL4-3_ and HIV-1_89.6_ consisting of 13 mismatches, the lowest genetic distance was 6 mismatches (seen in 4/10 possible pairs of the 5 HIV-1 strains) enabling the investigation of *in vitro* recombination (figure 2, orange bars).

### *In vitro* Recombination Frequency is Influenced by RT-PCR Conditions

PCR conditions can influence the formation of artificial chimeras [21], [22], [27]. To test the effect of *in vitro* recombination during RT-PCR, a 5-virus-mix was generated consisting of HIV-1_HXB2_, HIV-1_NL4-3_, HIV-1_JR-CSF_, HIV-1_89.6_, and HIV-1_YU2_. The molecular HIV-1 clones were mixed in approximate same amounts. In each of these and the following experiments, the exact same volume of the 5-virus-mix, the equivalent of 100,000 HIV-1 RNA copies, was used. After viral RNA was extracted, it was reverse transcribed using three different RT enzymes: Transcriptor High Fidelity RT, M-MuLV RT, RNase H^−^, and SuperScript III RT. Each cDNA synthesis was performed in duplicate. cDNA was amplified by outer and inner PCRs and pyrosequenced (table 2). A total of 1,649–100,263 reads were obtained per sample and artificial recombination was estimated using Recco. We found 30.6–37.1% of all reads to be artificial recombinants when the cDNA was amplified using standard PCR conditions and no adjustment of input copy numbers for nested PCR was performed (table 3, PR1-2).

**Table 2:** Detailed amplification conditions.

	NGS	PCR-NGS	RT-2PCR-NGS	PR1+ PR2	PR3 - PR8
			standard RT-PCR conditions	standard RT-PCR conditions	optimized RT-PCRconditions
**HIV-1 strains**	–––––– HIV-1_JR-CSF_ ––––––			––––––––––––– 5-virus-mix^a^ –––––––––––––	
**cDNA synthesis**					
input RNA copies	–	–	∼40,000	∼30,000	∼35,000
RT enzyme	–	–	Transcriptor RT	Transcriptor High Fidelity RT	Transcriptor High Fidelity RT (PR3+4)
					M-MuLV RT, RNase H^−^ (PR5+6)
					SuperScript III RT (PR7+8)
**1^st^ (outer) PCR**					
input cDNA copies	–	–	n.p.	n.p.^b^	∼10,000
dNTPs (mM)	–	–	0.2	0.2	0.4
oligonucleotides (µM each)	–	–	0.4	0.4	1
FastStart High Fidelity DNA polymerase (U)	–	–	1.25	1.25	3
denaturation 94°C (sec)	–	–	15	15	30
annealing 55°C (sec)	–	–	30	30	60
elongation 72°C (sec)	–	–	30	30	60
PCR cylces	–	–	30	30	30
final extention 72°C (min)	–	–	8	8	none
PCR product purification	–	–	yes	yes	yes
**2^nd^ (inner) PCR**					
input DNA copies	–	–	n.p.^c^	n.p.^c^	100,000
dNTPs (mM)	–	0.2	0.2	0.2	0.4
oligonucleotieds (µM each)	–	0.4	0.4	0.4	1
FastStart High Fidelity DNA polymerase (U)	–	1.25	1.25	1.25	3
denaturation 94°C (sec)	–	15	15	15	30
annealing 55°C (sec)	–	30	30	30	60
elongation 72°C (sec)	–	30	30	30	60
final extention 72°C (min)	–	8	8	8	none
PCR cylces	–	40	40	40	35

All concentrations are given per reaction.

n.p. qPCR was not performed.

a 5-virus-mix consists of the HIV-1 strains JR-CSF, NL4-3, HXB2, YU2 and 89.6 (see also Materials and Methods).

b 3/23 µl of cDNA was used for the 1^st^ PCR reaction, this corresponds to ∼4,200 cDNA copies.

c 10/50 µl of purified, undiluted 1^st^ PCR product was transferred to the 2^nd^ PCR.

**Table 3:** Frequencies of true and false haplotypes.

		Template				Estimated frequencies of truehaplotypes (%)						Estimated frequencies of falsehaplotypes (%)		
		copies/reaction				ShoRAH						ShoRAH		Recco
Sample	RT enzyme^a^	outerPCR	innerPCR	RT-PCRconditions^a^	Total reads/clonesanalyzed	HIV-1_HXB2_	HIV-1_NL4-3_	HIV-1_JR-CSF_	HIV-1_YU2_	HIV-1_89.6_	Sum	*in vitro*recombinants	Erroneoushaplotypes	*in vitro*recombinants
PR1	Transcriptor HighFidelity RT	n.p.	n.p.	standard	84,645	2.1	9.2	9.3	1.9	5.0	27.6	53.6	18.8	37.1
PR2	Transcriptor HighFidelity RT	n.p.	n.p.	standard	20,133	2.8	11.2	9.1	2.3	3.1	28.5	43.9	27.6	30.6
PR3	Transcriptor HighFidelity RT	∼10,000	100,000	optimized	3,846	6.2	39.6	24.2	10.9	17.4	98.3	0.3	1.4	0.9
PR4	Transcriptor HighFidelity RT	∼10,000	100,000	optimized	3,781	6.8	32.9	25.7	13.2	19.9	98.5	0.7	0.8	1.1
PR5	M-MuLV RT, RNase H^−^	∼10,000	100,000	optimized	14,482	4.5	45.4	22.9	11.4	11.7	95.9	1.2	2.9	1.2
PR6	M-MuLV RT, RNase H^−^	∼10,000	100,000	optimized	11,809	6.2	38.9	23.2	11.6	13.8	93.7	3	3.3	2.6
PR7	SuperScript III RT	∼10,000	100,000	optimized	2,046	5.3	40.4	26.4	9.6	17.4	99.1	0.5	0.4	1.7
PR8	SuperScript III RT	∼10,000	100,000	optimized	14,629	4.9	44.1	23.0	11.1	13.4	96.5	0.9	2.6	0.9
SGA1	Transcriptor RT	0.2	n.a.	n.a.	168	9.5	36.1	27.8	11.8	14.8	100.0	0.0	0.0	–
SGA2	Transcriptor HighFidelity RT	0.2	n.a.	n.a.	156	5.8	37.0	32.5	13.0	11.7	100.0	0.0	0.0	–
SGA3	M-MuLV RT, RNase H^−^	0.2	n.a.	n.a.	148	12.4	42.1	23.4	11.7	10.3	100.0	0.0	0.0	–

a see also table 2.

n.p. qPCR was not performed.

n.a. not applicable.

To reduce the high *in vitro* recombination frequency, PCR cycling conditions were optimized by increasing the elongation time to minimize the occurrence of prematurely terminated extension events [32], increasing dNTP and oligonucleotide concentrations, and omitting the final extension step [27] (table 2). Furthermore, after the first PCR, amplicons were quantified and 10^5^ DNA copies were transferred to the second round of amplification. With these modifications, the artificial recombination rate was reduced to 0.9–2.6% (table 3, PR3-8), as measured in six independent samples. The choice of the reverse transcriptase did not influence the artificial recombination rate.

### Characteristics of False Haplotypes Induced by *in vitro* Recombination

**Figure 3:**
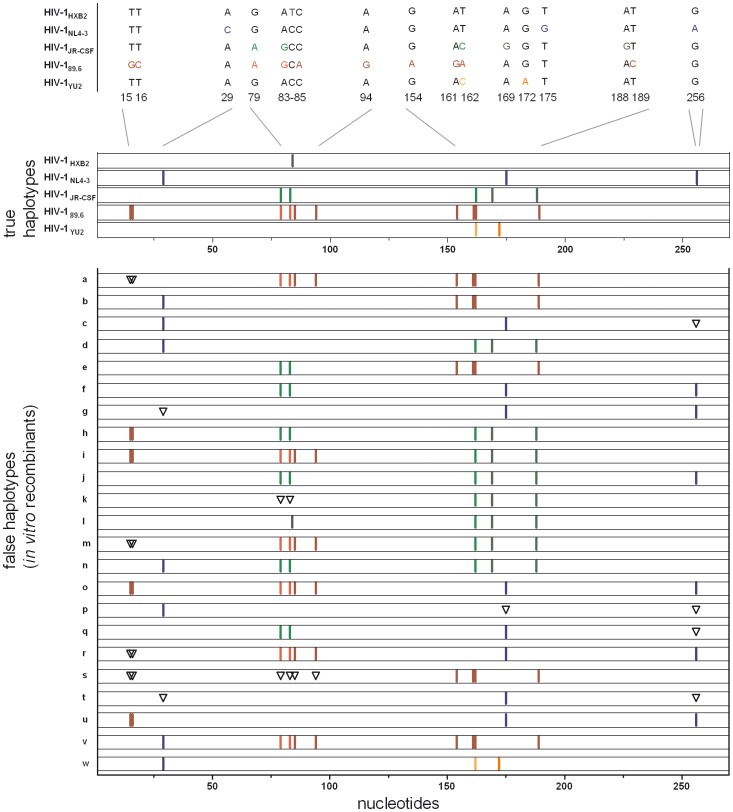
Major *in vitro* recombinant haplotypes assigned by ShoRAH. Haplotypes were aligned to the five reference strains and characterized. The top part shows the five virus strains (true haplotypes) of the 5-virus-mix and the bars indicate the specific mutation for each strain distinguishing it from the other four virus strains. The corresponding nucleotides and positions are indicated. HIV-1_HXB2_ has one unique mutation at position 84 (reference numbering 2362) that is indicated in grey. The mutations for HIV-1_NL4-3_ are marked in blue, in HIV-1_JR-CSF_ in green, in HIV-1_89.6_ in red, and in HIV-1_YU2_ in orange. Dark colours indicate unique mutations, light colours indicate differences to other strains but not unique for the respective strain. The bottom part shows all recombinant haplotypes found at 1% and higher frequencies. Triangles indicate positions were a specific nucleotide is expected according to the corresponding strain, but is missing. The Nucleotide positions in the sequences are indicated.

In the haplotypes reconstructed by ShoRAH, we observed the original strains together with other sequences. The latter can be subdivided in two classes: 1. recombinants of the strains (*in vitro* recombinants) and 2. viral variants harbouring artificial substitutions and/or indels (here called erroneous haplotypes). Applying standard RT-PCR conditions (samples PR1 and PR2), 53.6 and 43.9% of all reconstructed haplotypes, respectively, were classified as *in vitro* recombinants (table 3). These rates were substantially higher than the estimates of *in vitro* recombinants by Recco, which can be explained by the different algorithms and subsequent procedures used as explained in materials and methods. Figure 3 shows the 23 *in vitro* recombinants found at frequencies ≥1% of the viral population in samples PR1 and PR2, 17 of which could be found in both samples. Two recombination events per chimera can be clearly assigned to four *in vitro* recombinants (m, q, r and t, figure 3); for the remaining 19 *in vitro* recombinants, one recombination event can be clearly identified, although the occurrence of more than one recombination event cannot be excluded. For samples PR3-8, where optimized RT-PCR conditions were used, including limited input copy number, no *in vitro* recombinants were found at frequencies ≥1%. None of the 472 clones analyzed using single genome amplification was an *in vitro* recombinant. These results are consistent with the estimated recombination rates by Recco (table 3).

### Haplotype Reconstruction by ShoRAH Reveals Different Frequencies of True Haplotypes in Different Methodological Settings

ShoRAH was applied for haplotype reconstruction from 454 pyrosequencing data obtained from the 5-virus-mix containing HIV-1_HXB2_, HIV-1_NL4-3_, HIV-1_JR-CSF_, HIV-1_89.6_, and HIV-1_YU2_. The true haplotypes were successfully reconstructed in the following frequencies in these six samples obtained using optimized amplification conditions (PR3-8, table 3): 4.5–6.8% HIV-1_HXB2_, 32.9–45.4% HIV-1_NL4-3,_ 22.9–26.4% HIV-_1JR-CSF_, 9.6–13.2% HIV-1_YU2_, and 11.7–19.9% HIV-1_89.6_. Three independent single genome amplification experiments, using the same 5-virus-mix, resulted in similar frequencies of 5.8–12.4% HIV-1_HXB2_, 36.1–42.1% HIV-1_NL4-3,_ 23.4–32.5% HIV-1_JR-CSF_, 11.7–13.0% HIV-1_YU2_, and 10.3–14.8% HIV-1_89.6_ (table 3).

Haplotype frequency analysis based on 454 pyrosequencing data obtained using standard amplification conditions showed that less than 30% of all reads belong to the true haplotypes (PR1 and PR2, table 3). This leads to a substantial underestimation of the frequency of each true haplotype as compared to the proportions obtained by single genome amplification or 454 pyrosequencing from samples amplified applying optimized conditions (table 3).

## Discussion

Characterizing the diversity and evolutionary dynamics of virus populations within infected hosts is of great importance. For instance, it provides insights into virus escape mechanisms and development of drug resistance. Haplotype determination can be of therapeutic relevance, because pre-existing minority drug-resistant variants present in a patient can increase the risk of therapy failure as shown for HIV-1 in a recent meta-analysis [8]. NGS technologies enable the fast acquisition of thousands to millions of sequences from one sample, making it a powerful tool to study diverse virus populations. However, the analysis can be hampered by several experimental errors, occurring both during library preparation and sequencing (reviewed in [11]). For data analysis, two major *in vitro* artifacts have to be considered: 1) substitution and indel errors and 2) *in vitro* recombinants. Both can lead to wrong estimates of diversity within a virus population. This artificial diversity is difficult to distinguish from the real diversity especially for HIV-1, as the mutation rate is high and recombination also occurs frequently *in vivo* [33].

We designed a control experiment to estimate the substitution and indel error rates at each different amplification step of 454 pyrosequencing and of the sequencing technology itself. We used the same HIV-1 full-length plasmid, obtained from a single bacterial clone, and processed it with different techniques to estimate the error rate at each step of the pyrosequencing technology. The indel rates did not differ substantially between the different steps and occurred predominantly in homopolymeric regions clearly showing that these errors were generated during the pyrosequencing step [12], [34].

The substitution rate was >10-fold higher as previously described for non-pre-amplified genomic DNA fragments [12]. This might reflect the substitutions introduced by bacteria during the numerous duplications of the transfected plasmid. We expected that the substitution rate is higher in the samples generated by RT-PCR followed by outer and inner PCRs than in samples amplified only once prior to NGS. Interestingly, the substitution rates were only marginally higher in the PCR-NGS approach. Again, it might be possible that the overall diversity of plasmids obtained from bacteria after numerous duplications leads to a substantial amount of plasmids, which will not result in the generation of intact virus particles in transfected 293T cells. Thus, the transfection and the harvest of cell-free supernatant would display a bottleneck resulting in a less heterogeneous virus population compared to the plasmid “population”. In summary, our RT-2PCR-NGS experimental procedure showed an average substitution rate of ∼0.1%, which is consistent with previous studies [13], [35]–[39].

*In vitro* recombination represents a more severe problem especially for haplotype analysis of amplified viral RNA genomes. In our set-up, mixing five diverse virus strains, using standard amplification procedures and applying a very strict analysis by Recco, the *in vitro* recombination frequency reached up to 37%. Analysis by ShoRAH and manual inspection revealed an *in vitro* recombination frequency of up to 53.6%. These numbers may still underestimate the real *in vitro* recombination frequency, since up to 27.6% of false haplotypes were not clearly classifiable in these samples. Artificial chimeras inflate viral diversity estimates and, on the other hand, lead to wrong frequency estimates of the true haplotypes. Optimizing the amplification conditions and limiting the input DNA copy numbers in the second, outer PCR reduced the *in vitro* recombination rate to 0.9–2.6%. It has been previously shown that the input DNA copy number is a critical factor in the generation of artificial recombinants [19]. Despite these optimized conditions, our *in vitro* recombination rates were still higher than previously reported rates of 0.11–0.89% also using optimized PCR conditions [25], [28], [33], [38]. One major dissimilarity between our experimental approach and those methods was the template used. We performed a RT-PCR starting with HIV-1 RNA whereas the RT step was omitted in the other approaches, i.e., they started with viral DNA. It is known that prematurely terminated amplicons during PCR are mainly responsible for *in vitro* recombination in PCR reactions [20], [22], [25]–[27], [36], [40], [41]. However, in RT-PCR procedures, prematurely terminated cDNA fragments during cDNA synthesis can additionally serve as primers in subsequent amplifications [29]. Fang *et al.* showed that the *in vitro* recombination rate was approximately 3-fold higher when RT-PCR was compared to PCR alone (6.49% vs 2.65%, respectively) [29]. An *in vitro* recombination rate of 1.56%, i.e., more comparable to ours, has been recently published applying RT-PCR to generate amplicons for subsequent NGS procedures [42]. In a recent publication, Jabara *et al*. showed that by using degenerated primers for cDNA synthesis, PCR artifacts can be excluded from further analysis [16]. This approach is useful to exclude polymerase-induced misincorporations and PCR-induced recombination; however, it cannot identify RT-induced errors and recombinants.

It must be noted that our approach to amplify an almost equal mixture of five divergent virus strains is an extreme example of a heterogeneous population that enhances the likelihood of detectable recombination events. If the population is homogeneous, one will not find artificial chimeras to such an extent, but this does not mean that *in vitro* recombination does not occur. Rather, it means that one cannot identify any recombination event, because such an event can only be seen when it occurs between two different DNA molecules that can be distinguished by their genetic dissimilarity. In fact, a PCR-generated recombinant can only be the third most frequent haplotype if it is a chimera of the two most abundant ones [22]. On the other hand, the HIV-1 population of an infected individual is expected to show the classical quasispecies profile, with one dominant master sequence and a large number of low-abundant haplotypes. Thus, *in vitro* recombination between different haplotypes will be less evident. Nevertheless, it is important to know the possible *in vitro* recombination frequency within a sample, for example, when investigating superinfections.

The RT step in our experiments did not particularly affect the recombination rates. One reason could be that rather a short amplicon (290 bp) was amplified. Amplicon length can influence recombination rates. Two studies reported a low PCR-induced recombination rate (below 1%) within a short amplicon (120–265 bp) [26], [28]. Amplification of a longer amplicon (1.5 kb) led to higher recombination rates [43]. Fang et al. showed that *in vitro* recombination rates were higher in RT-PCR than PCR alone when amplifying a long molecule of over 4 kb [29]. The reasons might be lower efficiencies of reverse transcriptases to generate long amplicons, or that RNA is less stable than DNA, i.e., degraded RNA can lead to incomplete cDNAs. Amplicon sizes are increasing as a result of enhanced NGS read lengths; consequently, RT-PCR artifacts might become more abundant.

In summary, we have developed an optimized RT-PCR protocol suitable to amplify and sequence HIV-1 RNA genomes via 454 pyrosequencing exhibiting low error rates. We show that abundant *in vitro* recombinants influence haplotype reconstruction and lead to artificially high diversity as well as to a bias in quantification of true haplotypes present in the viral population. Thus, it is crucial to estimate and minimize the *in vitro* recombination frequency as well as to consider PCR- and RT-induced artificial errors in any subsequent analysis in terms of characteristics and frequencies of variants, especially those of RNA sources.

## Materials and Methods

### Viruses

Virus stocks were generated by separate transfection of 293T cells with each of the following subtype B HIV-1 full-length plasmids using Lipofectamine^TM^2000 (Invitrogen) according to the manufacturer’s protocol. The HIV-1 full-length plasmids were obtained through the NIH AIDS Research and Reference Reagent Program, Division of AIDS, NIAID, NIH: pYK-JRCSF from Irvin S.Y. Chen and Yoshio Koyanagi; pNL4-3 from Malcolm Martin; pYU2 from Beatrice Hahn and George M. Shaw, p89.6 from Ronald G. Collman, and pHXB2 was kindly provided by Marek Fischer. 48 h post transfection, virus-containing supernatant was collected and centrifuged for 5 min at 3,000 rpm and then filtered through 0.22 µm Sterilflip® (Millipore) to obtain cell-free virus stocks. For the 5-virus-mix, approximate same amounts of these molecular clones were mixed based on HIV-1 RNA copy numbers estimated by quantitative real-time PCR (qPCR). Viral stocks and mixtures were stored at −80°C.

### RNA Isolation, cDNA Synthesis, and Amplification of HIV-1 Protease Gene

10^4^–10^7^ HIV-1 RNA copies of each HIV-1 stock or 10^5^ HIV-1 RNA copies of the 5-virus-mix were used to isolate viral RNA using the NucleoSpin® RNA Virus Kit (Macherey and Nagel) according to the manufacturer’s protocol. HIV-1 RNA was treated with 5 U DNase (DNase I recombinant, RNase-free; Roche) for 30 min at 25°C followed by inactivation for 15 min at 70°C. DNase-treated RNA was reverse transcribed using an HIV-1 specific oligonucleotide RT pol 2787 5′-GTTCTCTGAAATCTAC-3′ (2772–2787 nt; this and the following oligonucleotide positions are based on HIV-1_HXB2_, GenBank accession number K03455) and different reverse transcriptases (RT) following the manufacturers’ protocols: Transcriptor Reverse Transcriptase (Roche), Transcriptor High Fidelity cDNA Synthesis Kit (Roche), M-MuLV Reverse Transcriptase, RNase H^−^ (Finnzymes), or SuperScript III RT (Invitrogen).

The outer PCR was performed using the FastStart High Fidelity PCR System (Roche), the forward oligonucleotide gag 2150 5′-AGCCAACAGCCCCACCAG-3′ (nt 2150–2167) (RT-2PCR-NGS and PR1+2, table 2) or gag 2142 5′- CAGACCAGAGCCAACAGC-3′ (nt 2142–2159) (PR3-8, table 2) and the reverse oligonucleotide pol 2727rc 5′-CTGGAGTATTGTATGGATTTTCAGG-3′ (nt 2703–2727) (RT-2PCR-NGS and PR1+2, table 2), or pol 2787rc 5′-GTTCTCTGAAATCTACTAATTTTCTCC-3′ (nt 2761–2787) (PR3-8, table 2). All oligonucleotides where synthesized by Microsynth. Detailed amplification protocols are given in table 2. The PCR product was purified using the NucleoSpin® Extract PCR purification Kit (Macherey and Nagel) according to the manufacturer’s description. Inner PCR was performed using forward oligonucleotide PR primer A 5′-CGTATCGCCTCCCTCGCGCCA-TCAG-MID-ATCACTCTTTGGCARCGACC-3′ and reverse oligonucleotide PR primer B 5′-CTATGCGCCTTGCCAGCCCGC-TCAG-MID-CCTGGCTTTAATTTTACTGGTACAG-3′ including A and B sequences at the 5′-ends necessary for the 454 FLX/Titanium pyrosequencing method, respectively, different multiplex identifiers (MID) and HIV-1 specific parts (underlined; nt 2259–2279 and nt 2569–2593, respectively). The PCR products were purified using the NucleoSpin® Extract PCR purification Kit (Macherey and Nagel) or the Agencourt AMPure XP PCR purification Kit (Beckman Coulter) according to the manufacturers’ descriptions.

### cDNA/DNA Quantification

The cDNA was quantified by qPCR with the oligonucleotides gag 2142 5′-CAGACCAGAGCCAACAGC-3′ (nt 2142–2159) and pol 2787rc 5′-GTTCTCTGAAATCTACTAATTTTCTCC-3′ (nt 2761–2787). qPCR was performed using a real-time cycler ABI7500 (Applied Biosystems) as follows: 95°C-3′, 50×(94°C-15′′, 55°C-30′′, 72°C-30′′) followed by a melt curve analysis in a total volume of 20 µl containing 5 nM (ROX, Invitrogen), 1.5 mM MgCl_2_, 0.4 mM dNTPs, 0.2×SYBR Green (Invitrogen), 0.4 µM of each oligonucleotide and 0.5 U JumpStart Taq Polymerase (Sigma). A standard was comprised of a 10-fold dilution series of an equimolar mix of DNA molecules from each viral strain. The first (outer) PCR amplicons were quantified similarly except that oligonucleotides pol 2316 5′-GCTCTATTAGATACAGGAGCAG-3′ (nt 2316–2337) and pol 2593rc 5′-CCTGGCTTTAATTTTACTGGTACAG-3′ (nt 2569–2593) were used.

### Preparation of Adaptor Ligated HIV-1 Fragment

A restriction endonuclease digestion was performed with AhdI and BsrGI (New England Biolabs) using the HIV-1 full-length plasmid pYK-JRCSF. The 381 bp long fragment (2281–2661 nt) was purified (QIAquick Gel Extraction Kit, Qiagen). 5′-adaptor (5′-GCCTCCCTCGCGCCATCAG-MID-C-3′ plus 5′-phosphat–MID-CTGATGGCGCGAGGGAGGC-3′) and 3′-adaptor (5′-GCCTTGCCAGCCCGCTCAG-MID-3′ plus 5′-phosphate-GTAC-MID-CTGAGCGGGCTGGCAAGGC-3′) were ligated to the fragment using T4 DNA Ligase (New England Biolabs). Adaptor-ligated fragments were gel purified.

### 454 Pyrosequencing

DNA was measured using the Quant-iT™ PicoGreen® dsDNA Assay (Invitrogen) according to the manufacturer’s protocol. Equimolar DNA amounts were pooled for emulsion PCR. Pyrosequencing was performed with the GS FLX System using the GS FLX Titanium MV emPCR Kit (Lib-A) or the GS Junior System using the GS Junior Titanium emPCR Kit (Lib-A) (Roche-454 Life Sciences).

### Single Genome Amplification

The frequencies of the five virus strains in the 5-virus-mix sample used for the standard PCR protocol were investigated using single genome amplification. cDNAs generated with the three RT enzymes Transcriptor RT, Transcriptor High Fidelity cDNA Synthesis Kit, M-MuLV RT, RNase H^−^, was diluted in such a way that each amplicon was derived from one cDNA copy (a maximum of 1 of 5 PCR reactions were positive). qPCR was performed in a real-time cycler ABI7500 (Applied Biosystems) as follows: 94°C-5′, 50×(94°C-15′′, 55°C-30′′, 72°C-45′′) followed by a melt curve analysis in a total volume of 20 µl containing 5 nM (ROX, Invitrogen) 1.5 mM MgCl_2_, 0.4 mM dNTPs, 0.1×SYBR Green (Invitrogen), 0.4 µM of the oligonucleotides gag 2023 5′-GGCTGTTGGAAATGTGGAAAGG-3′ (nt 2023–2044) and pol 2593rc 5′-CCTGGCTTTAATTTTACTGGTACAG-3′ (nt 2569–2593), and 1 U JumpStart Taq Polymerase (Sigma). Nucleotide sequence analysis of single genome amplicons were performed using the BigDye® Terminator v1.1 Cycle Sequencing Kit and an ABI3130 sequencer (Applied Biosystems).

### Data Analysis

The analyses of error rates for samples NGS, PCR-NGS and RT-2PCR-NGS were done separately for forward reads and reverse reads and restricted to the region nt 2281–2569 (based on HIV-1_HXB2_) that overlaps in all three experimental set ups. Substitutions, insertions and deletions were analyzed by aligning reads to the HIV-1_JR-CSF_ reference sequence after removal of reads with gaps of >10 nt. The alignments were computed with needle (implementing the Needleman-Wunsch algorithm), from the software suite EMBOSS [44] and the differences were counted as errors. Analysis of PR1-8 was done on the region nt 2279–2549 (based on HIV-1_HXB2_). Haplotype reconstruction was performed using the software ShoRAH (Short Read Assembly into Haplotypes) [45], a tool developed to correct sequencing errors in order to reconstruct the true local variants present in the virus population. ShoRAH clusters the reads (it groups them according to their similarity) and removes the intra-cluster variation to eliminate sequencing errors. The recombination analysis was performed using the software Recco [46]. Each read was included in a multiple sequence alignment with the five reference sequences (the viral strains in the 5-virus-mix) computed with muscle [47] and then passed to Recco. This software computes the number of “savings”, i.e., number of mismatches saved when explaining the read by a recombination event between two viral strains and mutations, rather than by a single strain and mutations only. For each sample, a histogram was produced counting the number of reads with a given number of savings. The proportion of recombinant reads was estimated on each sample by comparing the results obtained on the real reads to those results of a set of simulated reads obtained from the viral strains by substitutions only. In this analysis, reads were defined as recombinant when Recco assigns a savings value higher than two, which was the maximum value observed on the simulated reads. It is important to say that this analysis tends to underestimate the number of recombinant reads. In fact, even on datasets where all reads are recombinant, Recco reports saving values of two or less for a substantial fraction of the reads.
